# Activation of assembly factor for spindle microtubules triggers progression of renal cell carcinoma via Wnt3a pathway

**DOI:** 10.7150/jca.88063

**Published:** 2023-10-02

**Authors:** Zhijun Cao, Yu Li, Chen Xu, Zhiyu Zhang, Zhenfan Wang, Zheng Ma, Pengwei Xu, Xiaofei Sun, Xuefeng He, Jianglei Zhang, Hao Jiang, Gang Li

**Affiliations:** 1 Department of Urology, The First Affiliated Hospital of Soochow University, 215000, Suzhou, China.; 2 Department of Urology, Suzhou Ninth People's Hospital, Soochow University, 215000, China.

**Keywords:** ASPM, renal cell carcinoma, Wnt3a

## Abstract

Renal cell carcinoma, shorted as RCC is a well-known urological cancer with high level of morbidity and mortality. Although the regulatory role of the spindle microtubule assembly factor (ASPM) in tumor progression has been established, its relationship to the development of RCC remains unclear. To determine the significance of this gene in RCC, we examined its expression in RCC patients in the TCGA database and compared ASPM level between clinical samples of normal tissues and RCC tissues collected at our center. The prognostic relevance of ASPM was assessed by generating Kaplan-Meier survival curves and log-rank functions. Following alteration of ASPM expression using sh-ASPM or oe-ASPM transfection, RCC cell characteristics were evaluated through CCK-8, Transwell, and colony formation assays. Western blot analysis was conducted to measure levels of genes affected by ASPM, and rescue experiments were performed to explore the involvement of Wnt3a signaling in ASPM-mediated malignancy in RCC. Our findings indicate that ASPM is upregulated in RCC samples, and its levels are associated with the long-term survival of RCC patients. ASPM promotes the migration, proliferation, and invasiveness of RCC cells, and the Wnt3a pathway may be implicated in this process. In conclusion, these results indicate that ASPM contributes to the cancer progression of RCC by targeting the Wnt3a signaling pathway.

## Introduction

Renal cell carcinoma, shorted as RCC, is a prevalent urologic cancer that has increasing rates of morbidity and mortality. A study conducted in the United States showed that RCC had approximately 65,000 new cases and 15,000 deaths^1^. The predominant histologic classification of RCC is clear cell renal cell carcinoma (ccRcc), accounting for about 80% of RCC^2^. Poor progression of RCC is mainly attributed to metastasis. Generally, RCC responds unfavorably to chemotherapy as well as radiotherapy. Radiotherapy is the most effective treatment for metastatic renal cell carcinoma^3^, ^4^. However, early-stage metastasis in RCC limits surgical indications, leading to unsatisfactory clinical outcomes^5^. Despite numerous studies, the molecular mechanisms underlying RCC metastasis are still poorly understood. There is a pressing need to identify effective biomarkers for RCC metastasis at an early stage to improve patient outcomes.

Assembly factor for spindle microtubules (ASPM), initially discovered as a centrosomal protein influencing neurogenesis^6^, ^7^, has recently been found to play multiple roles in other processes like DNA damage repair, mitotic regulation, epithelial-mesenchymal transition and cell-cycle progression^8^-^10^. Studies suggest that ASPM can regulate Wnt signaling pathway activity in cancer cells, especially stem cells in hepatocellular, prostate, pancreatic, and gastric malignancy^11^-^14^. The maintenance of cancer stem cells (CSCs) by ASPM proteins occurs through the positive modulation of typical Wnt signaling pathways^15^. In hepatocellular carcinoma, ASPM was observed to positively regulate DVL1, an upregulating factor of Wnt signaling, which supports few "super potential subset of cancer stem cells (CSCs)"^11^. On the other hand, ASPM interacts with DVL2 in pancreatic cancer cells and influences it^7^, ^14^, suggesting that ASPM may influence DVL heterodimers in a specific manner. However, no investigations have investigated ASPM in the development of kidney cancer, specifically clear cell carcinoma, and its underlying mechanism. Recent advances in Targeted therapy have revolutionized the treatment landscape for advanced renal cancer. A notable breakthrough has been the identification and targeting of specific oncogenes that drive tumorigenesis and progression in renal cancer. For instance, the activation of the vascular endothelial growth factor (VEGF) pathway has been found to play a crucial role in angiogenesis and tumor growth. This knowledge has led to the development of VEGF inhibitors such as bevacizumab and sunitinib, which have shown promising results in clinical trials, leading to their approval for the treatment of advanced renal cancer^16^, ^17^.

This study aimed to investigate the impact of this gene on RCC. We evaluated ASPM expression levels in RCC patients from the TCGA database and compared them to clinical samples of RCC tissue and normal tissue collected at our center. The prognostic relevance of ASPM was assessed through Kaplan-Meier survival curves and log-rank functions. Following ASPM expression alteration using sh-ASPM or oe-ASPM transfection, we analyzed the characteristics of RCC cell by conducting colony formation assays, Transwell, and CCK-8.

## Materials and methods

### Database research

Bioinformatics analysis was conducted to explore ASPM in RCC as well as paraneoplastic tissues and their associated clinicopathological profiles, downloaded in The Cancer Genome Atlas (TCGA) database. The website of TCGA is https://cancergenome.nih.gov/. The edger function was used to analyze differences in expression levels between normal tissues and tumor, while survival function was employed to analyze patient prognosis. Following data processing, we determined the levels of ASPM and the survival rates of patients with RCC.

### RCC clinical samples

We collected 12 pairs of RCC and adjacent non-neoplastic tissues in the department of urology at the First Affiliated Hospital of Soochow University between March 2010 and September 2014. TNM staging was based on the system of Fuhrman histological grading. Patients were followed up until January 2019 for the purpose of tracking cancer recurrence, physical condition, and mortality via telephone and outpatient review. None of the patients underwent radiotherapy or chemotherapy before operation, and the samples that were collected were stored in liquid nitrogen. Informed consents were obtained from all participants, which met human subjects' criteria. This study was confirmed by the hospital's research and project ethics committee. The TNM system is a widely accepted staging system for various cancers, including RCC. It classifies tumors based on their Tumor size and invasion (T), involvement of Lymph Nodes (N), and presence of distant Metastasis (M).

### Cell culture and transfection

For cell transfection and culture experiments, RCC and epithelial cell lines (CAKI-1, OS-RC-2, ACHN, HK-2, 7860, 769P) were obtained from ATCC. RPMI-1640 (GIBCO, Carlsbad, USA) was used as cultures, except for CAKI-1 cells which were cultured in McCoy's 5A. 10% fetal bovine serum (FBS, GIBCO) and 1% penicillin/streptomycin (Invitrogen) were used and incubated in a humidified environment with 5% CO2 at 37°C. To overexpress ASPM and Wnt3a, complete cDNA sequences of ASPM and Wnt3a were inserted into the pLVX-puro vector to generate pLVX-Wnt3a and pLVX-ASPM; an empty vector served as the negative control. GeneChem (Shanghai, China) constructed the pLKO vectors containing ASPM or Wnt3a shRNA sequences. Lentiviral infections were performed on 769P, and CAKI-1 cell lines using lentiviruses containing sh-Wnt3a, phU6-EGFP-shRNA-ASPM, oe-ASPM, sh-NC, oe-Wnt3a, and oe-NC vectors. Stable transconductor pools were formed by puromycin (4 μg/mL) for two weeks.

### Q-PCR

Trizol reagent (Invitrogen, Carlsbad, CA, USA) was conducted to lyse cells and isolate RNA. The Primescript R Reagent Kit (Takara, Otsu, Japan) was used for reverse transcription of the qualified RNA into cDNA, followed by Premix Ex Taq (Takara, Japan) in qRT-PCR using SYBR Green. GAPDH was utilized as an internal reference. Each tissue was analyzed in triplicate, and relative levels were calculated using 2-ΔCt and normalized to GAPDH. Oligonucleotide primers were created employing Primer 7.1.2a as follows: ASPM: 5′-CCCCGACACCCGATGCCATTTG-3′ (forward) and 5′-TTAACCACCAAGTGAAGCCCTGTTC-3′ (reverse); GAPDH: 5′-CAGGAGGCATTGCTGATGAT-3′ (forward) and 5′-GAAGGCTGGGGCTCATTT-3′ (reverse).

### Western-Blotting

To carry out Western blotting, RCC cells and tissues were lysed with RIPA on ice for 20 minutes. The resultant mixture was centrifuged to isolate protein samples whose concentrations were determined by using the BCA method. To ensure uniformity, protein samples were adjusted to the same concentration, denatured, separated using SDS-PAGE, and transferred onto PVDF membranes. The membranes were then cut into strips based on molecular size and blocked in TBST containing 5% skim milk for 1.5 hours. After that, primary (1:500) and secondary (1:2000) antibodies were incubated, followed by ECL exposure and grayscale analysis using ImageJ software. The primary antibodies against ASPM and Wnt3a were sourced from Abcam, while Cell Signaling Technology supplied MMP2, GAPDH, MMP9, anti-rabbit, and anti-mouse secondary antibodies.

### CCK-8 assay

To perform the CCK-8 assay, 769P and CAKI-1 cells were added into 96-well plates at 3×10^3 cells/well. Optical density at 450 nm was measured using a CCK-8 kit (Dojindo Laboratories, Kumamoto, Japan) on days 1, 2, 3, and 4 to generate a viability curve for each sample.

### Colony formation

To perform the colony formation assay, 1×10^3 cells of 769P and CAKI-1 were seeded into each well of a 6-well plate. The medium was renewed once during the first week and twice during the second week. After two weeks, visible colonies were washed using PBS, fixed in methanol for 25 minutes, and stained with 0.1% crystal violet (purchased from Sigma-Aldrich) for another 25 minutes. Subsequently, the stained colonies were counted and visualized after being washed again in PBS.

### Transwell

The Transwell was performed to assess the invasive and migratory ability of cells. The experiment involved adding 300 microliters of suspension containing 2×10^4 cells to the top of Transwell inserts with an 8 μm pore size (Costar, Corning, NY, USA). In contrast, 500 microliters of medium containing 10% FBS were added to the bottom of the insert. After 48 hours of incubation, methanol treatment for 25 minutes and crystal violet staining for 25 minutes were used to induce and visualize migrating cells at the bottom of the chamber. Five random areas were selected per sample, captured using a microscope, and cell counts were determined. To assess invasive cells, Transwell chambers pre-coated with Matrigel (Invitrogen) were utilized, following the same procedure as mentioned above.

### Cell scratch assay

In this experiment, a confluent monolayer of cells is scratched via a sterile pipette tip to form a straight and uniform gap. The cells are then washed with medium to remove any detached cells and debris. Subsequently, the cells are incubated under normal culture conditions, and images of the scratch are captured at regular intervals using an inverted microscope equipped with a camera. The rate of cell migration is determined by measuring the degree of scratch closure over time. This assay can be used to evaluate the effect of different treatments or genetic modifications on cell migration in various cell types.

### Immunohistochemistry (IHC)

The ASPM protein was evaluated for positive expression in RCC tissues using the immunohistochemical technique. Tissue microarrays were first treated with a primary antibody and then incubated 24h at 4°C. This was followed by another day of HRP-conjugated antibody treatment and staining with diaminobenzidine. The IHC staining outcomes were independently appraised by two skilled pathologists. Based on these results, the RCC samples were classified into high-staining and low- clusters for further analysis.

### Statistical processing

The data analysis was performed using the SPSS 23.0 software package to ensure the accuracy of our statistical analysis. To evaluate the overall survival of renal cell carcinoma (RCC) patients, we utilized Kaplan-Meier survival curves. Additionally, we conducted Chi-square analysis to investigate any potential impact of ASPM on the clinical characteristics of RCC. To ensure the reliability of our findings, all experiments were independently conducted in triplicate. By replicating the experiments multiple times, we aimed to minimize any potential variability and increase the robustness of our results. In our statistical analysis, a p-value of less than 0.05 was considered statistically significant. This threshold allowed us to determine whether the observed differences between groups were likely due to chance or if they reflected true associations or effects. By employing these rigorous statistical analyses and experimental procedures, we aimed to enhance the validity and reliability of our study's conclusions regarding the relationship between ASPM and RCC.

## Results

### Phenotype and clinical value of ASPM in RCC

**Figure 1:**
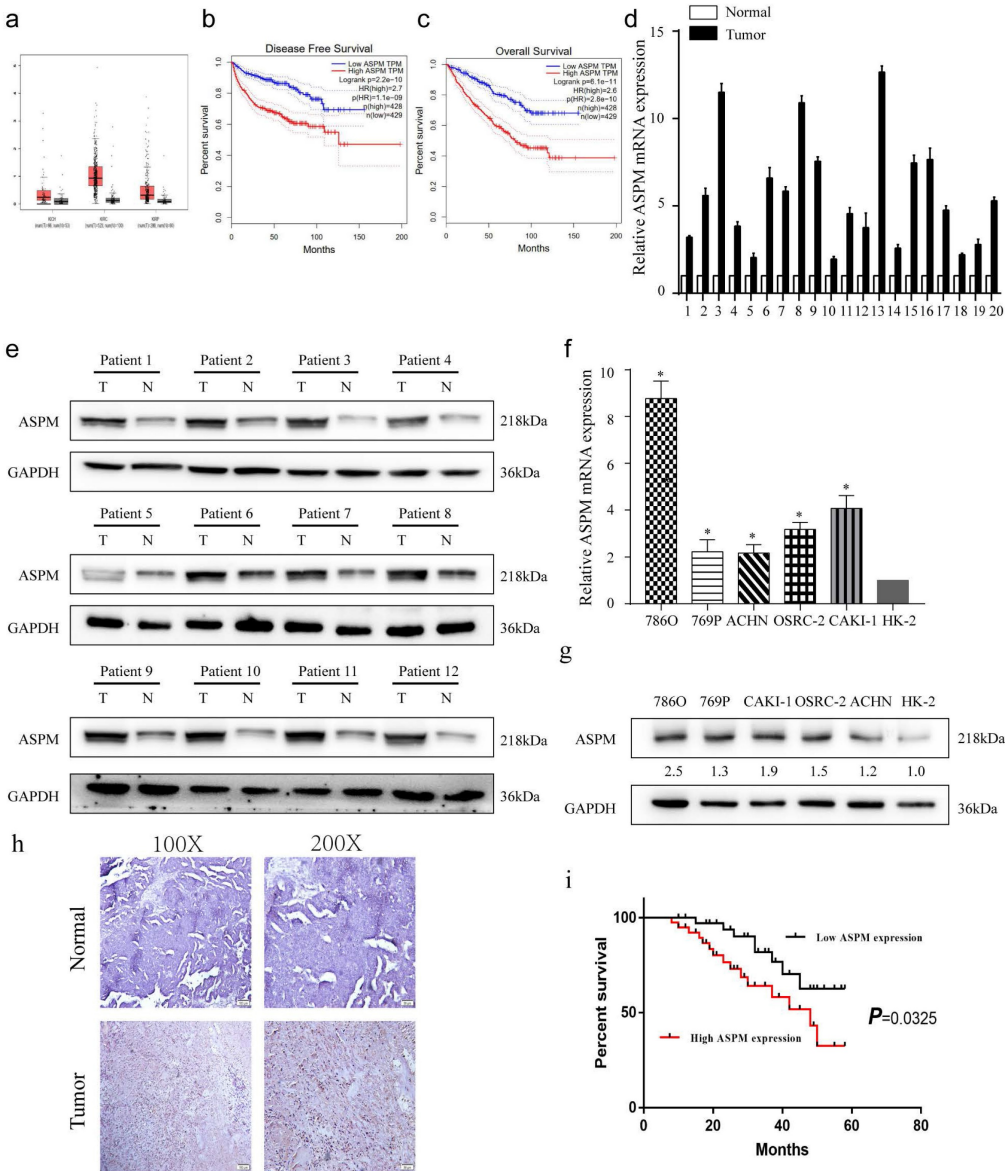
depicts the expression patterns of ASPM in RCC samples. Panel a show the levels of ASPM in normal and RCC tissues, as evidenced by TCGA profiles. Panels b and c present progression-free survival and overall survival curves for RCC patients classified according to high or low levels of ASPM in TCGA profiles. Panel d illustrates the differential levels of ASPM in 12 pairs of RCC and adjacent non-tumor tissues collected by our center. Panel e displays the protein levels of ASPM in 12 representative pairs of RCC and non-tumor tissues (T: RCC tissue, N: adjacent non-tumor tissue). Panel f shows the in vitro mRNA levels of ASPM in various RCC cell lines. Panel g presents the corresponding protein levels of ASPM in these RCC cell lines. Panel h comprises normal tissues and RCC tissues included in tissue microarrays evaluated via immunohistochemistry to determine relative protein levels of ASPM. Finally, panel i shows K-M survival curves based on ASPM levels in RCC patients. The symbol (*) signifies a statistically significant difference at P<0.05, while double asterisks (**) indicate a high level of statistical significance at P<0.01. The error bars represent the mean±SD values derived from t-tests.

From the TCGA database, a dataset containing 523 cases of patients with RCC, and 100 healthy controls was obtained. The analysis revealed that ASPM gene was increased in RCC patients, particularly in those with advanced tumors (Figure 1a, b). Additionally, a negative correlation was found between overall survival and high levels of ASPM expression in RCC patients (Figure 1c). Subsequently, we evaluated the expression profile of ASPM in RCC clinical samples obtained from our institution. In line with previous findings, the levels of ASPM were markedly higher in RCC samples (T) compared to adjacent non-tumorous tissues (N) (Figure 1d, e). Furthermore, ASPM expression was consistently elevated in vitro in RCC cell lines compared to control cells (Figure 1f, g). To validate the clinical relevance of ASPM, we performed IHC assays on tissue microarrays comprising 130 RCC cases, confirming that ASPM was overexpressed in RCC (Figure 1h). K-M curves further indicated that high ASPM expression could negatively impact the prognosis of RCC (Figure 1i). To determine the clinical significance of ASPM, we analyzed clinical data from RCC patients. Our observations displayed a positive association between ASPM expression and histological grade and TNM stage of RCC patients, but no association was observed with gender, age, histological subtype, and tumor size (Table 1).

### ASPM induces RCC proliferation in vitro

**Figure 2:**
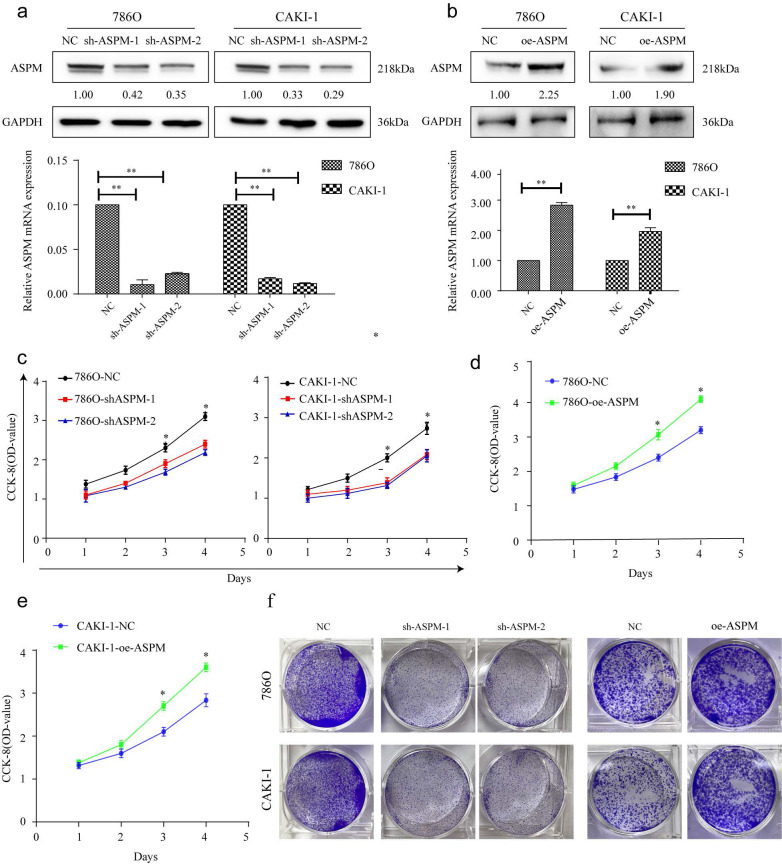
portrays the in vitro proliferation capability of ASPM-stimulated RCC. Panel a illustrates the transfection efficacy of sh-ASPM-1 and sh-ASPM-2 in CAKI-1 and 769P cells. Panel b shows the transfection effect of oe-ASPM in 769P and CAKI-1 cells. Panels d and e demonstrate the viability of 769P and CAKI-1 cells overexpressing ASPM, respectively. Panel f presents the colony formation of 769P and CAKI-1 cells knocked down by ASPM. The symbol (*) signifies a statistically significant difference at P<0.05, while double asterisks (**) indicate a high level of statistical significance at P<0.01. The error bars represent the mean±SD values derived from t-tests.

These experiments were conducted to evaluate the impact of ASPM on RCC cell proliferation. Among the various RCC cell lines tested, we selected 769P and CAKI-1 cells that displayed relatively high expression levels of ASPM for further investigations. Initially, we examined the knockdown effects of sh-ASPM-1 and sh-ASPM-2. Both shRNAs efficiently reduced the mRNA and protein levels of ASPM (Figure 2a). Similarly, the transfection efficiency of oe-ASPM was assessed by qRT-PCR and Western blot analysis (Figure 2b). The CCK-8 test demonstrated that silencing of ASPM significantly reduced the viability of both 769P and CAKI-1 cells, while overexpression of ASPM promoted cell proliferation (Figure 2c-e).

### ASPM stimulates RCC invasion and metastasis by modulating EMT

**Figure 3:**
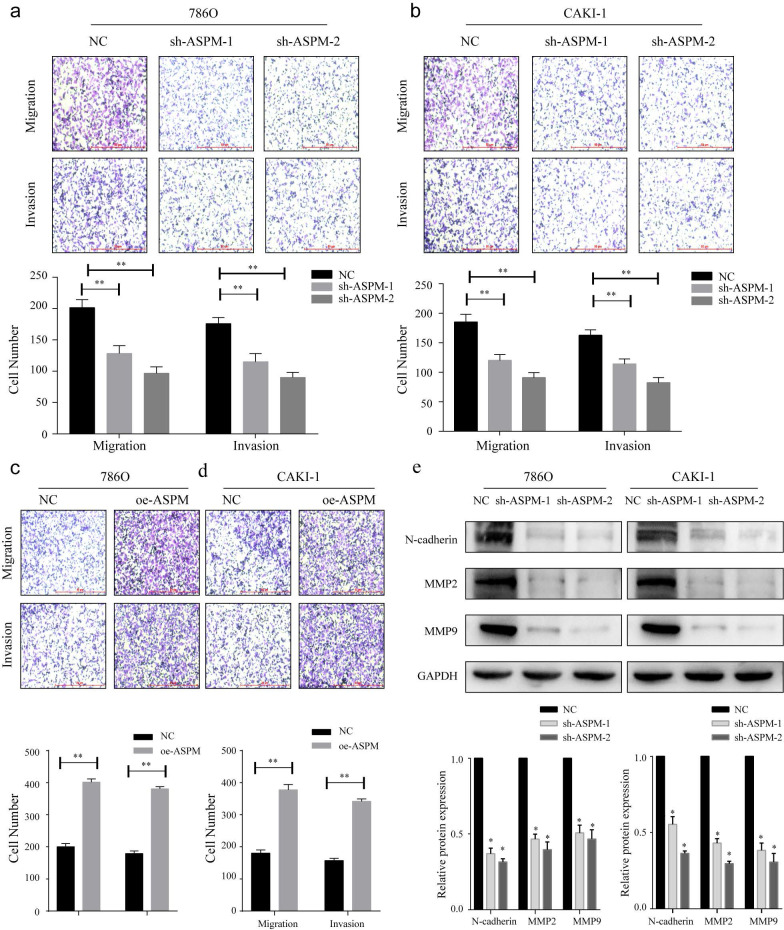
displays how ASPM can regulate EMT to stimulate the invasion and migration of RCC in vitro. Panels a and b depict the migration and invasion capabilities of 769P and CAKI-1 cells with ASPM knockdown, respectively. Panels c and d present the corresponding migration and invasion capabilities of 769P and CAKI-1 cells following ASPM overexpression, respectively. Panel e shows the protein levels of MMP2, β-catenin, and MMP9 in 769P and CAKI-1 cells cells that underwent ASPM knockdown. The symbol (*) signifies a statistically significant difference at P<0.05, while double asterisks (**) indicate a high level of statistical significance at P<0.01. The error bars represent the mean±SD values derived from t-tests.

To investigate the influence of ASPM on RCC invasion and metastasis, transwell assays were performed. In both 769P and CAKI-1 cells, shRNA-mediated knockdown of ASPM using sh-ASPM-1 or sh-ASPM-2 significantly decreased the number of migrating and invading RCC cells (Figure 3a, b). Moreover, overexpression of ASPM enhanced the invasive and migratory capabilities of RCC cells (Figure 3c, d). Furthermore, we assessed the relative expression levels of EMT proteins by Western blot analysis. Notably, the protein levels of MMP2, N-cadherin, and MMP9 were substantially downregulated in both CAKI-1 and 769P cells after knockdown of ASPM-1 (Figure 3e). Collectively, our findings indicate that ASPM modulates EMT to promote RCC migration and invasion.

### ASPM induces Wnt3a pathway to exacerbate RCC

**Figure 4:**
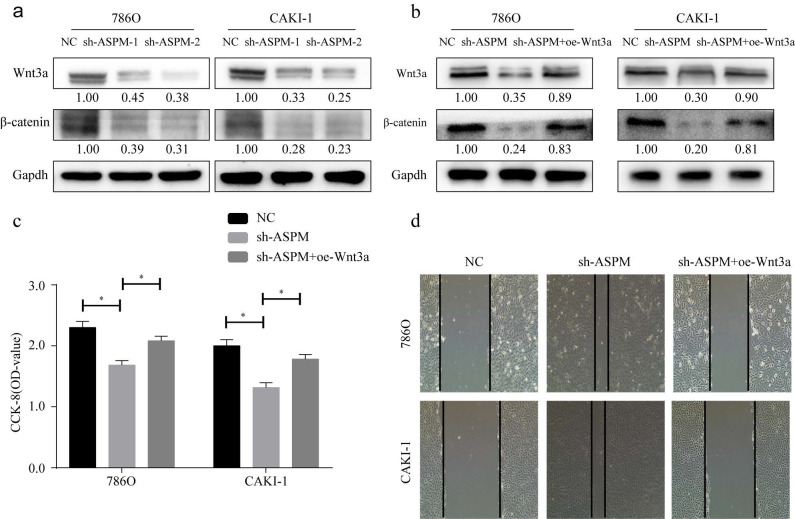
showcases the partial role of Wnt3a in the ASPM-induced deterioration of RCC. Panel a illustrates the protein levels of Wnt3a and β-catenin in 769P and CAKI-1 cells that underwent ASPM knockdown. Panel b presents the corresponding protein levels in 769P and CAKI-1 cells following ASPM overexpression. Panel c shows the CCK8 proliferation results of 769P and CAKI-1 cells affected by Wnt3a and ASPM. Finally, panel d demonstrates the scratch assay of 769P and CAKI-1 cells influenced by Wnt3a and ASPM. The symbol (*) signifies a statistically significant difference at P<0.05, while double asterisks (**) indicate a high level of statistical significance at P<0.01. The error bars represent the mean±SD values derived from t-tests.

Wnt3a signaling is closely related to EMT. In this study, we examined the protein of genes involved in Wnt3a signaling in 769P and CAKI-1 cells. As shown by Western-Blot analysis, silencing of ASPM significantly reduced the protein levels of β-catenin and Wnt3a (Figure 4a). In contrast, protein levels were elevated in samples overexpressing APSM (Figure 4b). In addition, we measured the proliferation of both cell lines by CCK8 and found that knockdown of ASPM diminished the proliferation of both kidney cancer cell lines, while some elevation in cell proliferation levels occurred if Wnt3a was overexpressed. Furthermore, using CCK8 assays, we evaluated the effect of ASPM on cell proliferation in both kidney cancer cell lines. Our data indicated that silencing of ASPM suppressed the proliferation of both cell lines, while overexpression of Wnt3a stimulated cell proliferation to some extent (Figure 4c). These findings were further corroborated by the results of the cell scratch assay (Figure 4d).

## Discussion

RCC is a prevalent and challenging cancer to treat, as current radiotherapy and chemotherapy options have limited effectiveness^18^. Therefore, targeted gene-based individualized therapy has emerged as an attractive approach in oncology treatment due to its precise and tailored efficacy. ASPM is frequently found to be expressed in the ventricular zone of the posterior cranial fossa, where it is known to regulate neurogenesis, mitotic spindle formation, and brain development^19^-^21^. Our investigation revealed that ASPM was highly expressed in RCC tissue samples collected from patients, and this finding was consistent with previous studies using downloaded RCC profiles. Notably, silencing ASPM significantly attenuated the proliferation, invasion, and migration abilities of 769P and CAKI-1 cells. Furthermore, we observed that ASPM intervention led to cell cycle arrest, which may be linked to the decreased proliferative capacity of RCC. Overall, our investigation indicate that ASPM serves as an oncogene involved in the pathogenesis of RCC. In RCC, studies have shown that ASPM is upregulated in tumor tissues compared to normal kidney tissue. Increased ASPM expression has been associated with higher tumor grade, advanced stage, and poorer prognosis in RCC patients. ASPM may promote tumor growth and metastasis by enhancing cell proliferation and migration and inhibiting programmed cell death.

ASPM is known for its high expression levels in many solid tumors, such as bladder cancer, breast cancer, prostate cancer, and CNS tumors^22^-^24^. Previous studies have shown that ASPM stimulates the progression and metastasis of prostate cancer through the Wnt-Dvl-3-b-catenin pathway^12^. In glioblastoma, it has been suggested that ASPM regulates Wnt-b-catenin signaling and G1 restriction point progression to promote tumor growth^25^. Kidney papillary cell carcinoma (KIRP) and kidney clear cell carcinoma (KIRC) are leading causes of tumor-related mortality^26^, ^27^, with no reliable treatment options currently available. In hepatocellular carcinoma of the liver (LIHC), there are currently no precise biomarkers available to predict response to immunotherapy^28^. ASPM was proposed as a potential biomarker for bladder cancer and a promising target for future immunotherapeutic strategies^29^. However, the relationship between clinical prognosis and immune cell infiltration and ASPM in other types of cancer remains unknown. ASPM is frequently overexpressed in many cancers and has been linked to a poor clinical prognosis and an increased risk of recurrence^30^. A growing body of evidence suggests that ASPM plays a critical role in regulating cell stemness, particularly in prostate cancer cells. Through its modulation of Wnt/b-catenin signaling, ASPM promotes the acquisition of a stem cell phenotype and enhances tumor aggressiveness^29^, ^31^. In bladder cancer, ASPM has been identified as an immune-related gene. However, it remains unclear whether ASPM is related to immune cell infiltration in other types of cancer. Notably, elevated expression of the ASPM gene has been associated with poor outcomes and increased aggressiveness in bladder tumor^7^. The lack of correlation between ASPM gene expression and histological subtypes of RCC in our study does not diminish the well-established association between clear cell carcinoma and poor prognosis. It highlights the need for further investigation and consideration of additional confounding factors when assessing the clinical significance of ASPM expression in RCC.

The classic Wnt/β-catenin pathway is comprised of three primary components: membrane proteins, disruption complexes (DCs), and β-catenin proteins. Frizzled receptors for the glycoprotein ligand Wnt and co-receptor Low Density Lipoprotein Receptor Related Protein 5/6 are in the cell membrane^32^. DCs consist of Axin, APC, CK1, and GSK-3^33^. The β-catenin protein serves as a critical hub within the Wnt/β-catenin pathway, influencing intracellular signaling and activating the transcription of many genes related to cellular activity^34^. Without extracellular Wnt ligands, β-catenin undergoes phosphorylation by DC composed of Axin, GSK-3, CK1, and APC. CK1 phosphorylates Ser45 of the β-catenin N-terminal structural domain, followed by sequential phosphorylation of Ser33, Thr41, and Ser37 by GSK-3^33^. Moreover, TCF/LEF family transcription factors interact with co-repressor Transducing-Like Enhancer/Groucho downstream of the Wnt/β-catenin pathway to suppress target gene transcription^35^. Dishevelled (DVL) genes are also critical components of the Wnt/β-catenin pathway. Binding of extracellular Wnt ligands to FZD-LRP5/6 can induce DVL to promote Axin binding to LRP5/6 in the WNT-FZD-LRP5/6 complex. This destabilizes DC stability and inhibits β-catenin degradation^36^. The cytoplasmic β-catenin concentration increases, and the protein is transported to the nucleus via binding with nucleoporin proteins^35^. Meanwhile, β-catenin interacts with LEF/TCF family members and coactivators to activate target gene transcription, like survivin, cyclin D1, and c-myc, while regulating differentiation, cell proliferation and apoptosis^37^. Our study revealed that ASPM may modulate the Wnt/β-catenin pathway, which correlates with changes in the above-mentioned transcription factors.

In conclusion, knockdown of ASPM resulted in significant downregulation of Wnt3a, β-catenin in RCC cells, while upregulating of ASPM produced the opposite trend. Moreover, Wnt3a was able to reverse the regulatory effect of ASPM on RCC cell phenotype. This suggests that ASPM exacerbates the malignant process of RCC by activating Wnt signaling.

## Figures and Tables

**Table 1:** Study Participant Characteristics at Enrollment

Variables	Total (n = 140)	Cohort, median (IQR)		Chi‐square d‐test p-value
		ASPM low (n = 70)	ASPM high (n = 70)	
Age, (years) n (%)				0.488
≤60	85 (61)	40 (57)	45 (64)	
>60	55 (39)	30 (43)	25 (36)	
Gender, n (%)				0.037
male	87 (62)	37 (53)	50 (71)	
femal	53 (38)	33 (47)	20 (29)	
Tumor size (cm), n (%)				0.735
≤4	102 (73)	50 (71)	52 (73)	
>4	38 (27)	20 (29)	18 (27)	
Histological subtypes, n (%)				1
clear cell carcinoma	121 (86)	60 (86)	61 (87)	
others	19 (14)	10 (14)	9 (13)	
Histological grade, n (%)				<0.01
I-II	110 (79)	65 (93)	45 (65)	
III-IV	30 (21)	5 (7)	25 (35)	
TNM staging, n (%)				<0.01
I	105 (75)	63 (90)	42 (60)	
II-IV	35 (25)	7 (10)	28 (40)	

## Declarations

### Availability of data and materials

All data and materials used in this study were available upon request.
